# Rehabilitation management for patients with spinal muscular atrophy: a review

**DOI:** 10.1186/s13023-025-03888-w

**Published:** 2025-07-10

**Authors:** Wei Song, Xiaohua Ke

**Affiliations:** Department of Rehabilitation Medicine, Shanghai Fourth People’s Hospital, School of Medicine, Tongji University, Shanghai, China

**Keywords:** Spinal muscular atrophy, Rehabilitation, Management

## Abstract

The rehabilitation management of patients with spinal muscular atrophy is a complex, multidisciplinary process aimed at slowing disease progression, preventing complications, and enhancing patients’ quality of life. Key components include motor function management, respiratory function support, swallowing function management, posture alignment, and the use of assistive devices. The various types of spinal muscular atrophy differ significantly in symptoms, progression rates, and severity, which poses unique challenges in rehabilitation management.

## Introduction

Spinal Muscular Atrophy (SMA) is a rare genetic neuromuscular disorder with autosomal recessive inheritance primarily characterized by the degeneration of motor neurons in the spinal cord, leading to progressive proximal muscle weakness and skeletal muscle atrophy [1]. In recent years, breakthrough advances in gene therapy—such as the development of nusinersen and risdiplam—have greatly improved treatment prospects for SMA [2]. These therapies aim to increase survival motor neuron (SMN) protein levels and have shown significant potential in slowing disease progression and enhancing survival, particularly with early intervention [3].

However, despite these advancements, SMA patients continue to face challenges with impaired motor function, respiratory difficulties, and limitations in daily activities, making rehabilitation management a critical component of SMA care. Rehabilitation has become a key element of clinical management, aiming to optimize functional abilities, enhance quality of life, and prevent secondary complications, such as joint contractures and respiratory depression. Recent research underscores the importance of a multidisciplinary approach [4], with rehabilitation strategies that encompass physical therapy, respiratory support, nutritional management, and psychological care. Physical therapy focuses on maintaining muscle strength and flexibility, while respiratory therapy is essential for managing breathing challenges, especially in the more severe forms of SMA.

This review aims to provide an overview of recent advancements in the rehabilitation management of SMA patients, emphasizing the importance of comprehensive rehabilitation assessment, treatment planning, and the role of emerging technologies—such as robotics and assistive devices—in enhancing rehabilitation outcomes. Rehabilitation strategies addressing SMA-related functional challenges are discussed through a summary of recent and ongoing studies.

## Epidemiology and pathophysiology of SMA

### Epidemiological trends in SMA

While SMA predominantly affects infants and children, it can also occur in adults. The incidence rate is approximately 1 in 10,000 to 20,000 live births, with a carrier frequency in the general population estimated at 1 in 40 to 1 in 70 individuals [5]. In Japan, an epidemiological survey as of December 2017 reported an estimated prevalence of 1.17 per 100,000 people and an incidence of 0.51 per 10,000 live births [6]. The incidence of SMA among newborns in China is 1 in 9,788, with a carrier frequency as high as 1 in 50 [7]. Approximately 90% of cases have onset during infancy, and 50% of affected children die before the age of two. It is estimated that there are around 30,000 to 50,000 individuals currently living with SMA in China, with about 1,200 new cases diagnosed each year [8]. The variability in SMA prevalence and carrier frequency across different countries underscores the need for global awareness and region-specific screening strategies. Differences in genetic background, diagnostic practices, and healthcare access all contribute to the observed disparities. Given that early diagnosis and intervention are critical to improving patient outcomes, it is imperative for policymakers and healthcare systems—particularly in countries with limited data—to invest in SMA screening programs, public education, and the establishment of national registries. Enhancing awareness and data collection will not only improve early detection but also support research and resource allocation tailored to local needs.

### Pathophysiology of SMA

SMA results from mutations in the SMN1 gene, which lead to a deficiency of the SMN protein, a protein essential for motor neuron maintenance [9]. SMA varies widely in severity, with the most severe form, SMA type I, often leading to significant movement impairment and respiratory failure, posing survival challenges within the first few years of life. SMA types II and III allow for longer life expectancy, but patients still experience serious mobility limitations and a reduced quality of life.

## Challenges for patients with SMA

### Economic burden of SMA

The economic burden of SMA on patients and their families encompasses direct, indirect, and informal care costs. Direct costs include expenses related to medical treatments, hospitalizations, and medications. A study on the economic burden of SMA in Spain found that the average annual costs per patient were €33,721, with direct healthcare costs accounting for €10,882 (around 32.3% of the total cost) and direct non - healthcare costs amounting to €22,839 (67.7% of the total cost) [10].

Patients with SMA and their families encounter numerous difficulties. The progressive nature of the disease leads to increasing physical limitations, which can result in challenges in performing daily activities, mobility, and self-care. Caregivers often face a heavy burden, including physical, emotional, and financial stress. In a study of caregivers of patients with neurodegenerative diseases, poor caregiver mental health was found to predict greater patient mortality, highlighting the impact of the caregiver’s well-being on the patient [11].

Communication between patients, families, and healthcare providers can also be a challenge. In some cases, patients may not feel comfortable speaking up about problems during their care, which can lead to unmet needs and lower satisfaction with the care received. A study of hospitalized patients found that many did not always feel comfortable speaking up about problems in their care, and these patients provided lower ratings of nurse and physician communication and overall hospital ratings [12]. In the context of SMA, effective communication is crucial for ensuring that patients and families understand the disease, treatment options, and available support services.

Funding programs can play a significant role in alleviating the financial burden on SMA patients and their families. Some governments and non-profit organizations offer grants or financial assistance to help cover the costs of treatments, medications, and rehabilitation services. For example, certain foundations may provide funding specifically for rare diseases like SMA to support research, patient care, and access to treatments [13].

Patient Advocacy Organizations (PAOs) also have an important role. They can raise awareness about SMA, advocate for better access to care and treatment, and provide support and resources to patients and families. However, there are concerns about potential conflicts of interest when PAOs have financial relationships with industry. A survey found that while most PAOs receive modest industry funding, a minority receive substantial support, which may raise questions about their independence [14]. Insurance companies can also impact the economic burden by providing coverage for SMA-related treatments and services. However, improving insurance coverage requires addressing issues such as transparency, pre-authorization processes, and ensuring that coverage is comprehensive and affordable. Additionally, exploring cost-effective alternatives, such as generic medications or home-based rehabilitation programs, can help reduce the financial burden on patients and families.

### Disparities in access to rehabilitation services

Disparities in access to rehabilitation services for SMA patients exist and can significantly impact their outcomes. A scoping review of disparities in rehabilitation following stroke found that there were disparities in access based on factors such as race, ethnicity, age, socioeconomic status, and urban/rural status [15]. Similar disparities may be present in SMA care. For example, patients from lower socioeconomic backgrounds may face financial barriers to accessing rehabilitation services, including the cost of therapy sessions, assistive devices, and transportation. Racial and ethnic minorities may also experience disparities in access, which could be due to factors such as differences in insurance coverage, provider availability, and cultural barriers. Addressing these disparities is crucial to ensure that all SMA patients have equal access to high-quality rehabilitation services. This may involve policy changes to improve insurance coverage, increasing the availability of rehabilitation providers in underserved areas, and providing culturally sensitive care.

### Disease progression and impact on motor function

Disease progression in SMA is characterized by a decline in motor function over time. In a cross-sectional study of 180 patients with SMA types I-IV, declining muscle strength and loss of motor skills were found to be characteristic of all SMA types, with the exception of the early phases in some children with SMA types II and III who may show temporary improvements [16]. In the referenced study, patients with SMA showed a progressive decline in motor function over time. On average, muscle strength decreased by at least 1 point per year on the Medical Research Council (MRC) scale, which assesses individual muscle groups, and functional motor abilities declined by approximately 0.5 point per year on the Hammersmith Functional Motor Scale– Expanded (HFMSE), reflecting reduced performance in gross motor tasks.

The age at loss of specific motor skills was associated with disease severity. For instance, triceps, deltoid, iliopsoas, and quadriceps were the weakest muscles in all patients. The progressive muscle weakness and loss of motor function start in childhood and continue into adulthood. This decline in motor function has a significant impact on the patients’ ability to perform daily activities, leading to increased dependence on caregivers and a reduced quality of life. Understanding the disease progression and its impact on motor function is crucial for developing appropriate rehabilitation strategies and therapeutic interventions to slow down or halt the decline.

## Rehabilitation management

**Fig. 1:**
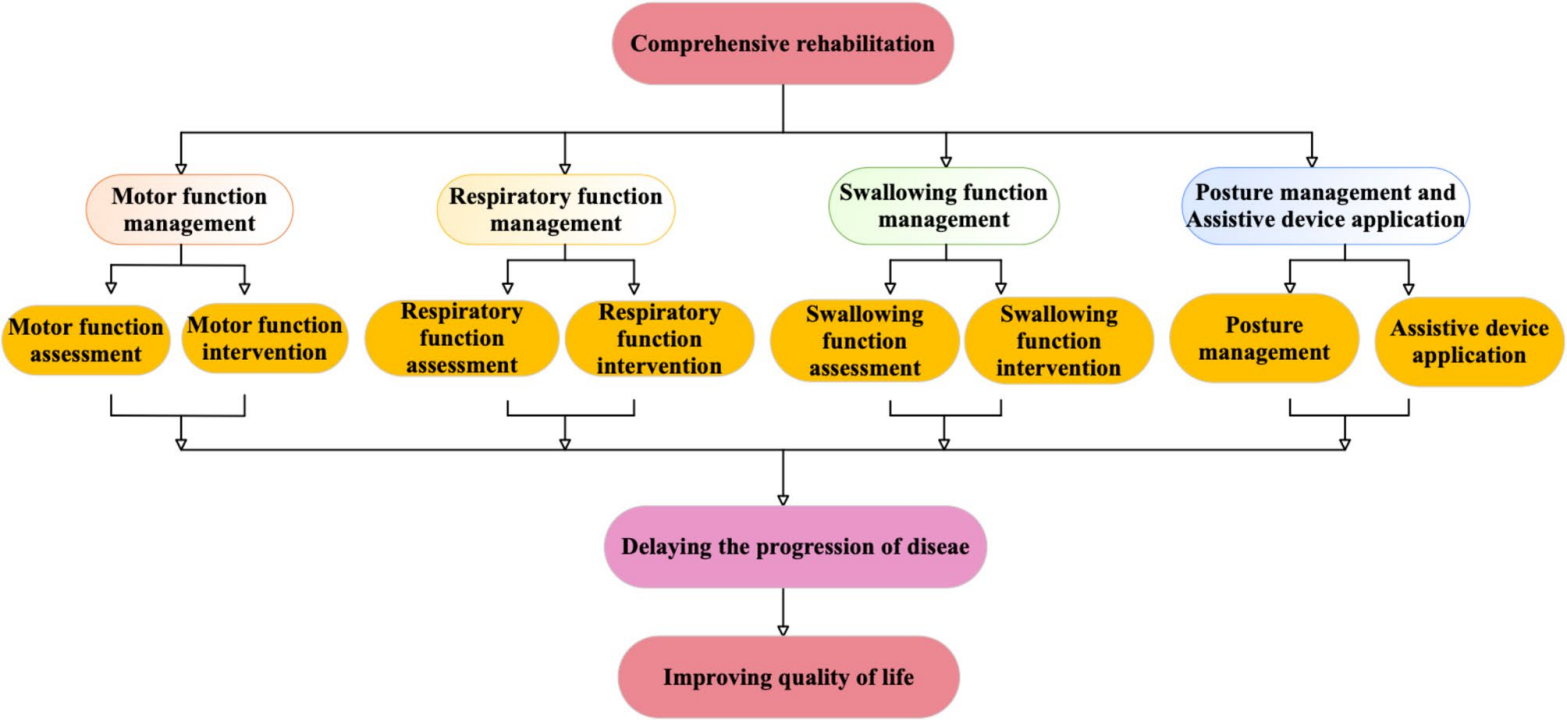
Comprehensive Rehabilitation Framework for Functional Management in SMA. (This diagram outlines a structured approach to comprehensive rehabilitation for individuals with SMA. The framework consists of four key domains: motor function management, respiratory function management, swallowing function management, and posture management with assistive device application. Each domain includes targeted assessment and intervention processes tailored to the progressive and multisystem nature of SMA. These rehabilitation strategies aim to delay the progression of disease and ultimately improve quality of life by preserving functional abilities, enhancing respiratory and nutritional status, and supporting independence through assistive technologies)

Long-term, standardized rehabilitation management is essential for SMA patients (see Fig. 1). It not only helps slow disease progression but also prevents or minimizes muscle atrophy, bone deformities, and enhances patients’ mental health and quality of life. With the clinical introduction of disease-modifying therapies, the rehabilitation management of SMA patients now faces both new challenges and opportunities.

### Motor function management

#### Motor function assessment

Regular functional assessments are essential for the formulation of individualized rehabilitation programs, the monitoring of disease progression, and the timely adjustment of therapeutic strategies in patients with SMA. Given the heterogeneity in motor abilities, muscle strength, and joint mobility across different SMA types and disease stages, the selection of appropriate assessment tools must be tailored to each patient’s age, clinical presentation, and functional status.

Assessment domains typically include gross and fine motor function, muscle strength, joint range of motion, respiratory status, and postural control [17]. Age-and function-specific tools are crucial, especially considering the evolving nature of SMA across the lifespan. For instance, in infants and young children with SMA type I or II, commonly used instruments include the Children’s Hospital of Philadelphia Infant Test of Neuromuscular Disorders (CHOP INTEND) [18] and the Hammersmith Infant Neurological Examination (HINE). For older children and ambulatory patients, the HFMSE, Revised Upper Limb Module (RULM), and the 6-Minute Walk Test (6MWT) are frequently applied to assess functional capacity and endurance [17]. The WHO Motor Developmental Milestones remains relevant for tracking early developmental progress. Muscle strength assessments are essential for tracking disease progression and guiding physiotherapy intensity. While manual muscle testing (MMT) is still commonly used in clinical settings, more objective methods such as hand-held dynamometry and quantitative muscle testing (QMT) are increasingly being adopted in clinical trials for better reliability and reproducibility. Furthermore, self-reported outcome measures such as the SMA Independence Scale (SMAIS) or the Pediatric Evaluation of Disability Inventory Computer Adaptive Test (PEDI-CAT) are being used to evaluate functional independence in daily life and help quantify changes meaningful to patients and caregivers. The regular application of these tools enables a comprehensive understanding of the patient’s functional trajectory and informs both therapeutic decisions and research outcomes.

#### Motor function intervention

The basic principles of neuromuscular rehabilitation in SMA revolve around addressing the muscle weakness and motor function impairment caused by the disease. One key principle is to enhance muscle strength and endurance through appropriate exercise programs. For instance, a study on a patient with spinal and bulbar muscular atrophy showed that a 15-week moderate-intensity exercise program combining weight-lifting and functional exercises led to meaningful performance improvements [19]. While encouraging, this was a single-case study, and thus its generalizability is limited. Nonetheless, it highlights the potential benefits of tailored exercise in motor neuron disorders.

Another principle is to improve motor coordination and postural control. Kanner described a case in which Therapeutic Scoliosis-Specific Exercises (PSSE) were used pre-and post-operatively in a child with SMA and scoliosis undergoing spinal fusion surgery [20]. Though based on a single case, the report provides useful insight into integrating PSSE to stabilize gross motor function and enhance post-surgical recovery, suggesting a promising adjunct to conventional rehabilitation.

SMA is caused by homozygous deletions or mutations in the SMN1 gene, resulting in reduced SMN protein levels and lower motor neuron degeneration [21]. The resultant denervation at the neuromuscular junction (NMJ) contributes to muscle atrophy. Hsieh et al. explored the mechanisms behind functional improvements in adults treated with nusinersen, identifying two key processes: reactivation of dormant motor neurons and collateral reinnervation through axonal sprouting [22]. This molecular understanding supports the rationale for early and intensive rehabilitation to complement pharmacological therapy. Muscle structural changes in SMA further complicate rehabilitation. Otto et al. conducted a longitudinal MRI study on untreated SMA patients and found increasing fat infiltration in thigh muscles despite unchanged strength score [23]. This underscores a disconnect between clinical scoring and underlying pathology, suggesting that early intervention—even before clinical decline—is vital to slow disease progression.

Motor function decline varies across SMA subtypes. Jira et al. showed that ambulatory SMA adults who were prone to falls had a significantly greater decrease in gait speed during the 6-minute walk test compared to non-fallers [24]. While the retrospective design limits causal inference, the study highlights the utility of gait metrics in identifying patients at high risk of functional decline, guiding timely rehabilitation strategies. However, the evidence base for physical therapy in SMA remains limited. Bartels et al. conducted a Cochrane review on combined strength and aerobic exercise for SMA type III and found the certainty of evidence to be very low due to small sample sizes and methodological heterogeneity [25]. Despite these limitations, cautious implementation of low-to-moderate intensity, patient-specific training may still be beneficial, especially when carefully monitored to avoid fatigue and overuse. In clinical practice, rehabilitation plans should consider patient-specific factors such as SMA types, disease severity, fatigue threshold, and treatment history. While high-quality trials are still needed, emerging evidence and clinical experience both support early, continuous, and individualized physical therapy to preserve function and enhance quality of life.

### Respiratory function management

#### Respiratory function assessment

Regular assessment of respiratory function is essential and should include evaluating cough strength, respiratory rate, chest shape, respiratory muscle strength, lung function, blood oxygen saturation, and nighttime breathing. Coughing is an important mechanism for clearing respiratory secretions, but respiratory muscle weakness in SMA patients can lead to insufficient cough strength and an inability to effectively clear secretions. SMA type I present severe respiratory muscle weakness at birth with reduced thoraco-abdominal movements, thoraco-abdominal asynchrony, tachypnea, and weak cough and crying. In children with SMA type II, Vital Capacity (VC)% predicted is already low at the age of 6–8 years and decreases thereafter by about 10% per year. VC is preserved in patients with SMA III until early adulthood with about a 5% decrease thereafter [26]. Regular measurements of maximum inspiratory pressure (MIP) and maximum expiratory pressure (MEP) can help assess respiratory muscle function. A study in adult SMA patients found that sniff nasal inspiratory pressure (SNIP) was a better predictor of non-invasive ventilation (NIV) needs compared to maximal inspiratory pressure (MIP), with an AUC of 0.84 for SNIP and 0.57 for MIP in predicting NIV initiation [27]. Oral function tests can detect subtle bulbar function impairments. A study comparing oral function in 58 persons with SMA and 45 healthy individuals found that non-ambulatory, untreated patients with SMA type II had lower scores in most oral function tests compared to controls, while ambulatory, treated patients with SMA type III had comparable strength and endurance values [28]. Changes in these values can serve as early indicators of respiratory failure, signaling the need for respiratory support or further intervention.

#### Respiratory function intervention

For patients with SMA and respiratory insufficiency, the timely initiation of non-invasive positive pressure ventilation (NIPPV) is a critical intervention [29]. Evidence from a randomized clinical trial has demonstrated the effectiveness of non-invasive respiratory support in neonatal populations, although the direct applicability to SMA patients remains limited due to population differences [29]. Respiratory muscle strength training is also essential for improving respiratory function in individuals with SMA [30]. A prospective cohort study reported a progressive decline in respiratory muscle strength over time in SMA patients, underscoring the importance of early clinical intervention [30]. In cases of weak cough ability, a comprehensive summary of airway clearance strategies used in neuromuscular disorders, such as Duchenne muscular dystrophy, provides valuable guidance for SMA management [31]. Techniques such as manual cough assistance—where a therapist applies abdominal or thoracic pressure to enhance cough effectiveness—can be implemented alongside other physical therapy interventions like postural drainage and vibration therapy. These measures aim to promote airway clearance and reduce the risk of pulmonary infections caused by secretion retention, a particularly important consideration for individuals with SMA type I.

### Swallowing function management

#### Swallowing function assessment

Swallowing disorder screening is essential for the early detection of swallowing difficulties in SMA patients. During screening, clinicians use questionnaires, simple tests, and reports from patients or family members. If screening results suggest a possible swallowing disorder [32], a comprehensive clinical swallowing function assessment should be conducted promptly. This assessment includes observing the patient’s oral and laryngeal function and evaluating their swallowing reflex, cough reflex, and throat-clearing ability [33]. Commonly used tools in clinical assessments include the water swallow test, fiberoptic endoscopic evaluation, and video fluoroscopic swallowing study. Regular swallowing function assessments for SMA patients, including initial screening and detailed clinical evaluation, are recommended [34]. This approach helps to identify potential swallowing issues early on and prevents complications like aspiration and malnutrition through timely interventions, ultimately improving patients’ quality of life.

#### Swallowing function intervention

Maintaining proper eating posture is essential for reducing the risk of aspiration and improving swallowing efficiency in individuals with SMA [35]. A study [35] investigating the effects of sitting posture and bolus volume on the activation of swallowing-related muscles—using surface electromyography in healthy adults—demonstrated that upright and forward-leaning postures can optimize muscle engagement during swallowing. While the participants were not SMA patients, the study’s controlled design and objective muscle activity measurements offer foundational evidence for posture-related recommendations in dysphagia management. However, caution is warranted when applying these findings to the SMA population, given their frequent muscle weakness and spinal deformities. For patients with dysphagia, the medical team or speech therapist typically provides guidance, often including posture adjustments tailored to the individual’s functional capacity. Commonly recommended positions include upright or semi-upright sitting, chin-tuck posture, or lateral lying. Additionally, modifying food consistency and texture—such as offering soft foods, thickened liquids, and smaller, more frequent meals—is an effective strategy to promote swallowing safety [36]. A Cochrane systematic review [36] summarized moderate-quality evidence supporting the use of modified food and fluid consistencies to reduce aspiration pneumonia risk in patients with dementia. Despite the differences in disease etiology, the underlying swallowing physiology shares similarities with neuromuscular dysphagia, thereby supporting the use of such interventions in SMA rehabilitation. Swallowing function training remains a core component of therapy, aiming to strengthen and coordinate relevant musculature. These exercises often target the lips, tongue, facial muscles, and laryngeal muscles, and may include repetitive swallowing drills. In severe cases where oral intake is unsafe, enteral nutrition via nasogastric tube or percutaneous endoscopic gastrostomy may be required [37]. A recent narrative review [37] summarized current approaches to nutritional and gastrointestinal management in children with SMA type I, providing expert recommendations and emphasizing the importance of multidisciplinary care. While the review’s methodological rigor is limited, it reinforces the need for individualized feeding strategies based on ongoing assessment. Personalized swallowing rehabilitation programs, continuously adjusted according to the patient’s evolving needs and clinical evaluations, can help preserve functional ability and potentially delay the progression of dysphagia.

### Posture management and assistive device application

#### Posture management

SMA patients often develop abnormal posture, muscle contractures, and bone deformities due to progressive muscle weakness, especially in supportive muscle groups [1]. To maintain proper body alignment and minimize musculoskeletal complications, posture management has become a cornerstone of SMA rehabilitation. This includes the use of orthoses, specialized seating systems, and standing frames [38]. When used appropriately, these devices can help preserve mobility, improve quality of life, and delay the progression of deformities.

Orthoses are external devices designed to support weakened muscles and skeletal structures, assisting patients in maintaining proper posture while preventing contractures and deformities [39]. Common types include spinal braces, lower limb orthoses, and hand or wrist splints. Prolonged improper sitting posture in SMA patients can lead to complications such as scoliosis, pelvic misalignment, and increased muscle tightness [40]. Properly adjusted seating systems and specialized chairs offer trunk support while enhancing independence in daily activities. As the disease progresses, some patients may require manual or powered wheelchairs for mobility. For those requiring prolonged sitting, custom seating systems contoured to the pelvis, spine, and lower limbs offer optimal support and encourage proper posture. Additional features, such as pelvic belts and leg supports help maintain alignment, reducing the risk of pelvic tilt or deformities like lower limb valgus or varus. Standing frames are also widely used in SMA rehabilitation to support upright positioning and standing exercises [41]. These devices provide multiple benefits, including promoting bone growth, preventing osteoporosis, reducing lower limb contractures, and improving circulation and digestion. Additionally, they help preserve muscle strength and cardiovascular health.

Posture management further plays a vital role in reducing joint stiffness and muscle contractures. While orthotics and related assistive devices primarily provide static support, they are often designed to allow a limited range of motion, enabling patients to perform minor active movements that prevent complications associated with prolonged immobilization. Regular adjustments of wheelchairs, standing frames, or bed positions help redistribute pressure, reduce strain on specific muscle groups, and minimize joint stress. For pressure-prone areas, pressure-relieving dressings or air mattresses can be used to alleviate localized pressure. Preventive skin care is essential for reducing the incidence of pressure ulcers and other skin-related issues in patients with SMA. It is recommended that patients be repositioned regularly—typically every two hours—to minimize prolonged pressure on the same area. In daily care, it is important to keep bed linens and clothing smooth and dry, avoiding wrinkles or dampness that could cause skin damage.

The dynamic position adjustments contribute to improved comfort and overall functional outcomes. The selection of equipment and frequency of adjustment should be tailored to each patient’s condition, including disease severity, muscle strength, skeletal structure, and skin integrity. Therefore, individualized customization of posture management tools is essential for optimizing rehabilitation management in SMA.

#### Assistive device application

Beyond posture management, assistive technologies play a key role in enhancing autonomy and quality of life of SMA patients. An internet of things (IoT)-based assistive system was evaluated in a child with SMA type I. The system, composed of an M5Stack Core2 kit, a mobile app, and smart switches, enabled the child to control smart devices within the rooms. The results showed that the control function of smart switches was the most used, and the usability scores obtained from both the patient and caregiver were 87.5% and 90%, respectively, with an average performance of the entire system at 93.33% [42]. These findings underscore the potential of smart assistive systems in empowering SMA patients with greater environmental control and independence in daily life.

Gait-assisted exoskeletons are also being explored for children with SMA. A systematic review focuses on their use in children with cerebral palsy and SMA, and the findings highlight the promise of this technology in enhancing walking ability across both conditions [43]. Additionally, the use of assistive technologies is associated with better quality of life scores in SMA patients. A cross-sectional study in China found that assistive technology was independently associated with a lower score in a negative direction in the Pediatric Quality of Life Inventory (PedsQL™), suggesting that it is beneficial for SMA patients [44]. These technologies can help SMA patients overcome physical limitations and participate more actively in daily life.

With the rapid advancement of artificial intelligence (AI), there is growing potential to develop more intelligent, responsive, and personalized assistive technologies for individuals with SMA. AI-powered systems could enable more intuitive control of devices through voice, eye-tracking, or even brain-computer interfaces, further reducing the physical barriers faced by patients [45]. These innovations not only support daily functional activities, but also facilitate social interaction, education, and communication, helping patients stay more connected with the outside world. Moving forward, it is hoped that more research institutions, technology companies, and healthcare providers will collaborate to design and produce smart assistive solutions tailored to the unique needs of SMA patients—ultimately improving their autonomy, well-being, and quality of life.

## Comprehensive management of multidisciplinary teams

The rehabilitation management of SMA patients is a complex, long-term process that requires expertise and skills from multiple fields [46]. As SMA is a progressive neuromuscular disease affecting motor function, respiratory function, swallowing, nutritional status, and mental health, a single department or specialty alone often can’t address the full range of patient needs. Collaboration within a multidisciplinary team (MDT) is essential for the rehabilitation management of SMA patients, ensuring they receive comprehensive, personalized assessment and treatment, which optimizes outcomes and enhances quality of life [47].

Neurologists play a central role in the diagnosis and management of SMA patients [4]. As the primary care providers for SMA, they are responsible for confirming the diagnosis, monitoring disease progression, assessing changes in neurological function, and developing drug treatment plans based on the latest advancements (such as gene therapy and other novel treatment options) [48]. To date, three novel gene therapy drugs, nusinersen, onasemnogene aberparvovec and risdiplam, have received marketing authorisation for SMA treatment by several authorities including Food and Drug Administration and European Medicines Agency [49]. Gene therapy is typically led by neurologists, who are responsible for developing the treatment plan, including selecting appropriate gene therapy drugs, determining the timing and dosage of treatment, and more. During the therapy process, neurologists also need to closely monitor the patient’s neurological responses and any potential complications. Neurologists also work closely with the rehabilitation team to regularly assess patients’ neuromuscular function and adjust treatment plans as needed.

Rehabilitation physicians are essential in the rehabilitation management of SMA patients. They focus on designing and overseeing individualized rehabilitation plans and coordinating various therapies. Their main goal is to help patients maintain maximum independence and quality of life through functional training, the use of orthopedic devices, and assistance with daily activities. Physical therapists are key to the motor function rehabilitation of SMA patients. They develop and implement training to improve muscle strength, range of motion, and posture control. The goal of physical therapy is to help patients maintain optimal motor function by strengthening muscles, enhancing movement patterns, and preventing complications such as joint contractures and bone deformities. Physical therapists also manage respiratory function, guide the use of non-invasive positive pressure ventilation, and provide respiratory muscle training and coughing techniques to prevent respiratory complications. Importantly, rehabilitation professionals must also adjust therapeutic plans before and after gene therapy. Prior to gene therapy, they assess the patient’s functional status and provide supportive care to optimize the therapeutic window. Following gene therapy, therapists play a crucial role in helping patients achieve maximal functional gains by modifying rehabilitation strategies to reflect improvements in muscle strength or endurance, and by addressing new therapeutic goals that emerge with functional recovery. This adaptive and individualized approach ensures that gene therapy outcomes are maximized through targeted rehabilitation efforts.

Nutritional management is crucial for SMA patients, who often experience swallowing difficulties, eating challenges, and muscle wasting. Nutritionists develop personalized plans based on evaluations of patients’ dietary intake, nutritional status, and weight changes, helping them maintain physical health and avoid issues like malnutrition or obesity.

Given that SMA is a chronic, progressive disease, patients and their families frequently face emotional challenges, including depression, anxiety, and psychological stress. Psychologists provide psychological support and interventions to help patients and their families manage these challenges, strengthen emotional resilience, and improve quality of life. In the rehabilitation management of SMA patients, single-discipline interventions are often insufficient to address the complex needs of these individuals [50]. The psychological aspect of SMA is significant, as patients and their families often face emotional challenges due to the chronic and progressive nature of the disease. Caregivers also face psychological burdens, including stress, anxiety, and depression, which can impact their well-being and the quality of care they provide. For example, in a study of caregivers of patients with neurodegenerative diseases, poor caregiver mental health was associated with greater patient mortality [11]. Addressing the psychological needs of SMA patients and their families is essential, and may involve psychological counseling, support groups, and education to help them cope with the emotional challenges of the disease.

Assessing the mental health of SMA patients and their families is crucial for providing appropriate support. Tools such as the Hospital Anxiety and Depression Scale (HADS) can be used to screen for anxiety and depression in patients and caregivers [51]. Strategies to help them cope with the difficulties of living with SMA include psychological counseling, which can provide emotional support, coping strategies, and help patients and families adjust to the changes brought about by the disease.

Support groups can also be beneficial, as they allow patients and families to share experiences, learn from others, and receive peer support. In addition, education about the disease, its progression, and available treatment options can empower patients and families to make informed decisions and take an active role in managing the disease. For example, providing information about the latest research and treatment developments can give patients and families hope and a sense of control over the situation.

**Fig. 2:**
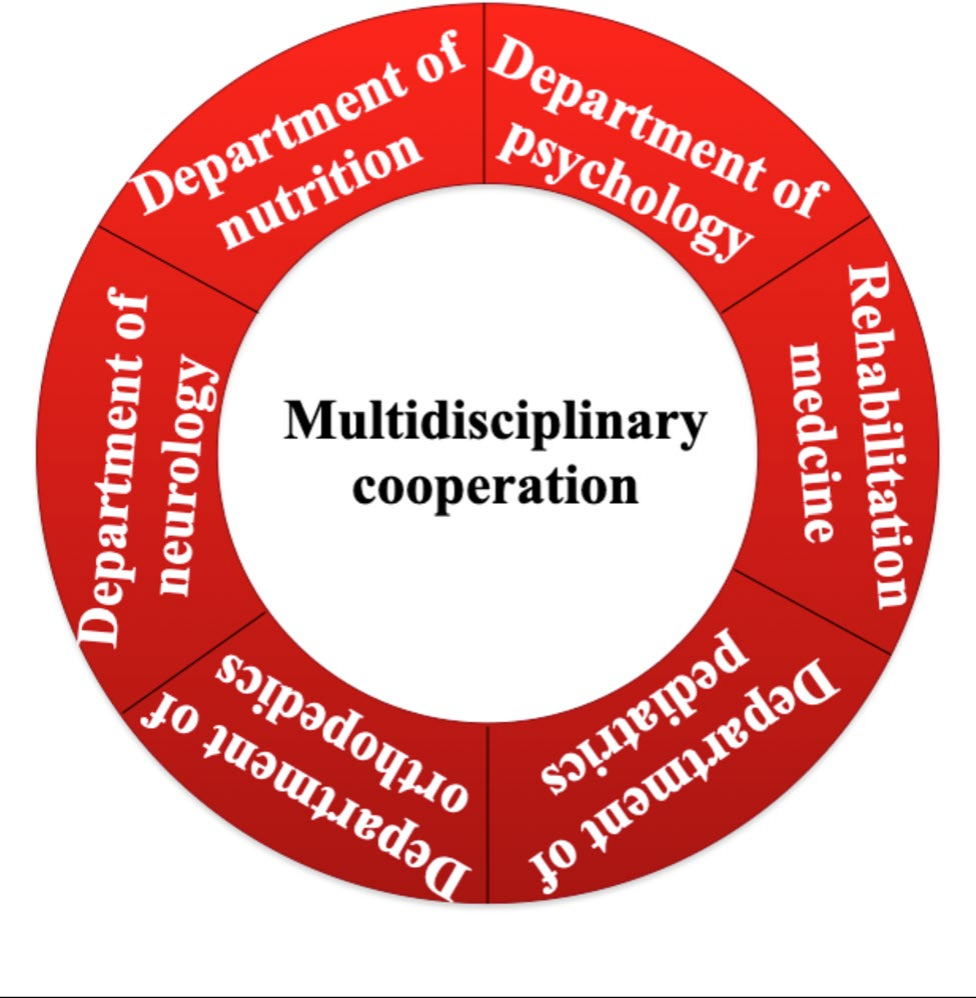
Multidisciplinary Cooperation Model in SMA Rehabilitation. (This diagram highlights the multidisciplinary collaboration required for effective rehabilitation in patients with SMA. The central concept of multidisciplinary cooperation integrates the expertise of various departments, including nutrition, psychology, rehabilitation medicine, pediatrics, orthopedics, and neurology. Each specialty contributes to comprehensive care strategies such as motor function training, respiratory management, nutritional support, psychological counseling, and orthopedic interventions. This coordinated approach ensures individualized and holistic treatment, aiming to optimize functional outcomes and improve quality of life for individuals with SMA)

A multidisciplinary team approach combines expertise from neurology, rehabilitation, nutrition, psychology, and other fields to create a more comprehensive and tailored rehabilitation plan (see Fig. 2). The team holds regular meetings to share assessments, discuss treatment options, and collaboratively develop and adjust personalized plans based on each patient’s unique situation.

## Future research directions and innovations in SMA rehabilitation

Future research in SMA rehabilitation should focus on several key areas. Firstly, there is a need to develop more effective and targeted rehabilitation interventions. This could involve further exploring the use of emerging technologies such as virtual reality and exoskeletons. For example, virtual reality-based rehabilitation could provide engaging and personalized exercise programs for SMA patients, potentially improving their motivation and adherence to rehabilitation [52]. Secondly, research should aim to better understand the long-term effects of disease-modifying treatments on rehabilitation. As more patients are treated with drugs like nusinersen, onasemnogene abeparvovec, and risdiplam, it is crucial to study how these treatments interact with rehabilitation over time. This includes investigating the optimal timing and intensity of rehabilitation in the context of long-term treatment. In addition, the application of AI in SMA rehabilitation holds great promise. AI-based systems could assist in designing individualized rehabilitation programs by analyzing large datasets on patient responses and functional progress. Machine learning algorithms may also help in predicting rehabilitation outcomes and optimizing therapy intensity and modalities for different SMA phenotypes.

Finally, neuromodulation is emerging as a promising avenue in the field of neurorehabilitation and may offer potential benefits for SMA patients. Techniques such as spinal cord stimulation (SCS) [53], transcranial magnetic stimulation (TMS) [54], and functional electrical stimulation (FES) [55] have shown positive effects on motor function and muscle activation in other neuromuscular disorders. While clinical evidence in SMA remains limited, early studies and preclinical findings suggest that neuromodulation could help enhance residual motor output, reduce fatigue, or slow functional decline when combined with traditional rehabilitation. Future research should explore the feasibility, safety, and long-term efficacy of neuromodulation techniques in SMA, particularly as adjuncts to physical therapy or pharmacologic treatments.

## Prospects

SMA is included in newborn screening programs worldwide [56]– [57], which presents a unique opportunity for early intervention. Early-identified patients can potentially benefit from rehabilitation strategies initiated at a younger age, when the body may be more responsive to treatment. For example, physical therapy started in the early stages can help prevent muscle atrophy and contractures, and promote the development of motor skills [58]. Multidisciplinary care, involving a team of neurologists, physical therapists, occupational therapists, speech therapists, and other specialists, can be tailored to the specific needs of these early-identified patients. Genetic counseling can also be provided to families, helping them understand the disease, its inheritance patterns, and available treatment options. By starting rehabilitation and multidisciplinary care early, the hope is to slow down the progression of the disease, improve the patient’s quality of life, and potentially achieve better long-term outcomes compared to patients diagnosed at a later stage.

However, different types of SMA present significant differences in symptoms, progression rates, and severity [59], which poses the challenges in creating standardized rehabilitation programs. In many regions, experienced rehabilitation teams and SMA-specific resources are lacking, resulting in inadequate access to appropriate services. Long-term adherence to rehabilitation training and equipment use is particularly crucial for SMA patients. However, maintaining compliance can be difficult due to factors such as fatigue, family burden, and time constraints. Additionally, the specialized equipment and prolonged care required for effective rehabilitation management are often expensive, making sustained treatment financially burdensome for many families [60].

There is also a scarcity of large-scale randomized controlled trials evaluating the long-term effects of various rehabilitation intervention, which limits the availability of evidence-based guidance. As our understanding of SMA subtypes and their progression improves, the development of more personalized rehabilitation programs tailored to the individual needs becomes increasingly feasible. Advances in telemedicine and mobile applications may allow patients to access remote guidance and monitoring, reducing the need for frequent clinic visits while maintaining rehabilitation outcomes. In the future, optimizing collaboration among multidisciplinary teams and improving cross-disciplinary efficiency will be essential for delivering comprehensive rehabilitation services. Furthermore, the development of innovative rehabilitation equipment and assistive technologies—such as robotic rehabilitation and virtual reality training—will play a vital role in helping SMA patients maintain or recover their functional abilities. More randomized controlled trials and long-term follow-up studies are also needed to validate the effectiveness of these interventions and strengthen the scientific foundation of SMA rehabilitation management.

## Conclusion

The rehabilitation management of patients with spinal muscular atrophy is a long and complex process that requires the collaborative participation and ongoing efforts of a multidisciplinary team. Through effective rehabilitation management, disease progression can be delayed, complications can be prevented, and patients’ quality of life can be enhanced. In the future, as disease-modifying therapies (DMTs) continue to develop and be implemented, the rehabilitation management of SMA patients will encounter both new opportunities and challenges.

## Data Availability

Not applicable.
